# A method for measuring closed-loop latency in gaze-contingent rendering without extra equipment

**DOI:** 10.3758/s13428-025-02864-3

**Published:** 2025-12-03

**Authors:** Matt D. Anderson, Emily A. Cooper, Jorge Otero-Millan

**Affiliations:** 1 Herbert Wertheim School of Optometry & Vision Science, University of California, Berkeley, California USA; 2 Helen Wills Neuroscience Institute, University of California, Berkeley, California USA; 3 Department of Neurology, Johns Hopkins University, Baltimore, Maryland USA

**Keywords:** Gaze-contingent rendering, Foveated rendering, Eye tracking, Latency

## Abstract

In gaze-contingent rendering, the visual stimulus rendered on a display changes based on where the observer is looking. This technique allows researchers to achieve dynamic control over stimulus placement on the retina in the presence of eye movements and is often used to investigate how sensory processing and perception vary across the visual field. Precise stimulus placement using gaze-contingent rendering depends on minimizing the temporal latency between a change in the observer’s gaze position, measured using an eye tracker, and the corresponding change to the stimulus. This latency, however, can be challenging to measure reliably. Here, we present a simple method for measuring system latency that requires no additional hardware beyond the eye tracker and display, which are already part of the gaze-contingent system. Two small circles are rendered on the display to simulate the appearance of two pupils. The eye tracker is pointed towards the display to record both pupils simultaneously. One pupil is drawn based on a pre-determined trajectory, for example, moving up and down at a constant speed. The second pupil is “gaze-contingent”: it is drawn based on the measured position of the first pupil. The time-lag at which the position of the second pupil matches the first pupil gives the closed-loop latency of the entire system. To validate this method, we added artificial rendering delays to our system and produced measured latencies that precisely corresponded to predictions, given the refresh rate of the display. This method provides a simple, low-cost way of precisely quantifying gaze-contingent rendering latencies, with no additional hardware required.

**Supplementary Information:**

The online version contains supplementary material available at 10.3758/s13428-025-02864-3.

## Introduction

Where a visual stimulus appears in the visual field affects how it is processed. For example, it is well known that it is easier to read letters and discriminate the fine details of objects if they appear in the center of our vision, rather than the periphery (Rosenholtz, 2016). However, precise experimental studies of how perception varies across the visual field can be challenging to perform because the eyes are in constant motion. These eye movements complicate researchers’ ability to deliver stimuli to specific locations in the visual field. Indeed, humans perform an average of 3–4 saccades per second, relocating the center of gaze (Yarbus, 1967). Also, even when the eyes are seemingly at rest, tiny eye movements (microsaccades and slow drifts) occur continuously (Otero-Millan et al., 2008). In order to compensate for these eye movements, researchers can employ a technique called gaze-contingent rendering. Gaze-contingent rendering is the procedure of continuously updating the visual stimulus presented to an observer based on where they are looking. It requires an experimental setup with a digital display and an eye tracker capable of measuring changes in the observer’s gaze position on the display. This technique allows researchers to achieve experimental control over where in the visual field a stimulus appears or how much it moves on the retina in the presence of eye movements.

Gaze-contingent rendering has been used in various areas of behavioral research. In research on retinal disease, for example, it offers a powerful tool for simulating visual field defects (artificial scotomas) in observers with normal vision (N. Chen et al., 2019; Kwon et al., 2013). In reading research, this technique has been used to explore how foveal, parafoveal, and peripheral areas of the retina contribute to word recognition (Rayner, 2014). And in studies of fixational eye movements, it has been used to cancel or control the retinal motion caused by slow eye drifts (Ağaoğlu et al., 2018; D’Angelo et al., 2024; Rucci & Victor, 2015), providing insight into why humans execute, and how we compensate for, eye movements during fixation. Gaze-contingent rendering is also applied in commercial virtual reality (VR) display systems because high-resolution imagery can be costly to render in real-time (e.g., Meta Quest series, Ross et al., 2023). By exploiting known deficiencies in human vision (e.g., the decline in visual acuity in peripheral vision) and varying image resolution across the visual field, manufacturers can improve device performance, potentially enhancing user comfort and enjoyment.

One critical variable that determines the quality of gaze-contingent rendering is the *closed-loop latency*: the delay between a change in the observer’s gaze position and a corresponding change in the rendered stimulus. In display systems with non-zero latency (that is, all real systems), a gaze-contingent stimulus will slip across the retina by an amount proportional to the speed of the observer’s eye movement and this closed-loop latency. Since the goal of gaze-contingent rendering is to control where in the visual field an image appears, and since researchers typically cannot control the speed of the observer’s eye movements, a core goal in applications of this technique is to minimize system latency. Prior research suggests that humans can tolerate an average of ~ 60 ms of latency before reporting awareness of gaze-contingent image manipulation (Albert et al., 2017; Loschky & Wolverton, 2007). The frame-to-frame variability in latency, which can be caused by fluctuations in graphics rendering demands and adaptive synchronization of the display refresh rate (used in display technologies like NVIDIA G-Sync and AMD FreeSync), may also impact perception, but this is less well researched. Precisely quantifying the distribution of system latencies and comparing these values to human thresholds is an important step for understanding and minimizing errors in gaze-contingency.

**Fig. 1:**
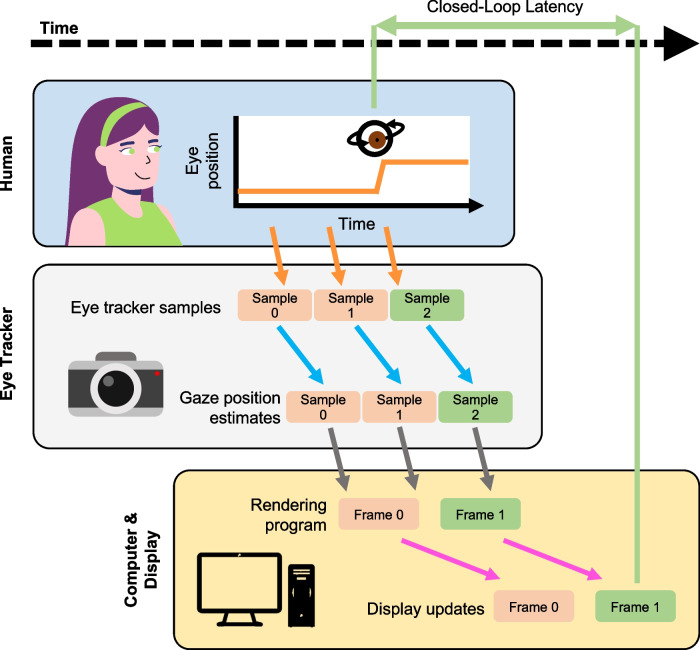
Sources of delays in a typical gaze-contingent rendering loop. Horizontal line lengths represent the approximate relative delay added by each process. The eye tracker captures the human observer’s eye position (*orange arrows*), and estimates the corresponding gaze position in screen coordinates (*blue arrows*). This information is transmitted to the display computer (*gray arrows*), and then the rendering program draws a new frame and updates the display (*pink arrows*). The *orange boxes* indicate measurements of gaze position where the eye is stationary, which results in no update to the stimulus. The *green boxes* indicate measurements taken when the eye moves. The time between the onset of eye motion and the corresponding update to the displayed stimulus (*green arrow*) is the closed-loop latency of the entire system

The closed-loop latency is the sum of multiple delays related to eye tracking image capture and processing, data transmission, stimulus rendering, and display updating. Figure 1 shows a diagram of a typical gaze-contingent rendering loop with these different delays. The loop begins the instant the eye moves, and ends when the on-screen stimulus is updated according to this movement. The first delay relates to the eye tracking system: once an image of the eye is captured (orange arrow), it is processed in order to obtain an estimate of the gaze position in display coordinates (blue arrow). Then, the resulting gaze position is transmitted to the computer to update the stimulus (gray arrow). Updating the stimulus on the display is a multi-stage process. First, the rendering program draws a fresh image to the frame buffer (a location in memory that represents the stimulus bitmap). The speed of this process is regulated by the complexity of the image and the capabilities of the graphics hardware (pink arrow). Then there is an additional delay between the command to update the frame and the display change, which is partly determined by the time each update is scheduled in the monitor refresh cycle (also pink arrow). The time that the new frame appears on the display marks the latency endpoint (green arrow).

Researchers have various methods available to them to quantify gaze-contingent rendering latencies. One potential method involves identifying all independent sources of delays in the rendering pipeline and adding them to arrive at a total system latency estimate. The drawback of this method is that sources of delays can be missed or hidden behind proprietary software. Another simple method is to assume that, as soon as a new gaze sample is obtained, the stimulus is updated on the next frame (Henderson et al., 1997; Loschky & McConkie, 2000; Loschky & Wolverton, 2007; Santini et al., 2007). The maximum possible latency according to this approach is the interval between two successive frame refreshes. This will only be a reasonably accurate approximation if the eye tracker sampling rate is significantly higher than the refresh rate of the monitor, if other sources of delays like the actual display change, gaze position estimation, and signal transmission are short enough, and if the display refresh rate is stable over time (not true for displays with adaptive synchronization). In most experimental setups, these assumptions are incorrect, which can be demonstrated using methods that leverage additional hardware.

One latency measurement approach requires attaching a photodiode to the display and measuring the delay between an eye-tracking event and a corresponding change in the on-screen luminance, typically with the help of a digital signal processor or an oscilloscope (Dorr & Bex, 2011; Ganesan et al., 2023; Stein et al., 2021). Photodiodes have a short rise time (as low as 1 ns), so this hardware can be used to measure latencies with high temporal precision. The disadvantage of this approach is that additional hardware setup is required, which can be logistically prohibitive and/or time-consuming. To address this problem, Saunders & Woods (2014) proposed an alternative version of this method that uses a relatively inexpensive commercial high-speed (1000 Hz) camera. Here, the experimenter shines a bright light into the eye-tracking sensor, effectively blinding it. At the precise moment when gaze tracking is lost, the rendering program changes the color/luminance of the on-screen stimulus. The entire setup is viewed from afar by a high-speed camera, and manual frame-by-frame analysis of the resulting videos is used to quantify the delay between light onset and display change. Generally, applications of methods that use additional measurement hardware have produced latency estimates that consistently exceed display refresh speeds (Dorr & Bex, 2011; Ganesan et al., 2023; Orlov & Bednarik, 2016; Saunders & Woods, 2014; Stein et al., 2021), highlighting the importance of empirical measurement.

**Fig. 2:**
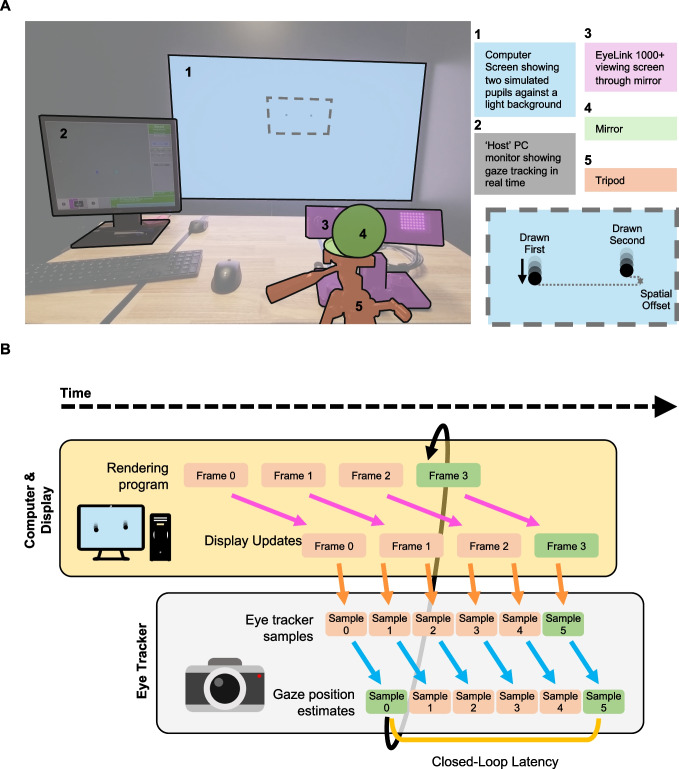
Experimental setup and latency estimation. **A** Setup and stimuli that were used to test our method. *Left*: Experimental setup; for a video of this setup in action, see Supplementary Video 1. *Bottom right*: cartoon visualization of moving pupil stimulus. The left pupil is drawn first, and the right pupil assumes a *y*-position based on the measured position of the left pupil. The latency creates a vertical spatial offset between the two pupils, which is used in our analyses. **B** Latency estimation using our method within the gaze-contingent rendering loop. The rendering program draws a pair of pupils and updates the display (*pink arrows*). Next, the eye-tracking camera takes a corresponding measurement of the pupil locations (*orange arrows*) and estimates the current gaze position (*blue arrows*). The vertical gaze position of the first (pre-determined) pupil is transmitted back to the rendering program (*black looping arrow*), and the second pupil is drawn at this location (while maintaining a constant lateral offset). Once the display is updated with the new pupil location, the eye tracker measures the gaze position again. The delay at which the measured *y*-position of the second pupil matches the first pupil (*green boxes*) gives the closed-loop latency (*yellow line*). This example assumes a fixed refresh rate

In this paper, we present a new method of estimating the closed-loop latency of gaze-contingent rendering. Our method resembles Saunders & Woods’ (2014) approach, but while they use a separate camera to detect the onset of on-screen events and record the latency of gaze-contingent stimulus changes, we use the eye tracker camera(s) to do both. The main requirements of our method are that the eye tracker needs to measure gaze binocularly and be capable of tracking simulated pupils presented on the stimulus display. Figure 2 illustrates how our method works. On a display, two laterally displaced black circles are rendered against a bright background to simulate the appearance of two pupils (Fig. 2A). We point the eye tracker towards the stimulus to record the position of both pupils simultaneously. One pupil moves up and down, at a constant speed, along a pre-determined path. As the eye tracker measures each new pupil position, the rendering program draws the second pupil at the measured *y*-location of the first pupil. Figure 2B illustrates the gaze-contingent rendering loop. The aforementioned delays of image capture (orange arrows), gaze position estimation (blue arrows), and signal transmission back to the display computer (black looping arrow), generate a time-lag between the measurement of the first pupil, and the corresponding update to the second pupil (green boxes). This, in turn, creates a spatial (vertical) offset between the two pupils, which the eye tracker measures. The time-lag at which the measured *y*-position of the second pupil matches the first pupil gives the closed-loop latency of the entire system (green line). This method requires no additional hardware and no manual annotation of the resulting data.

## Method

**Equipment.** This method requires components that almost any laboratory interested in gaze-contingent rendering will already use for the experiments: one or two computers, a display, and a high-speed video-based binocular eye tracker. In all the following tests, the display computer was a HP Z2 Tower G9 Workstation, with a 13th Gen Intel CPU and 32 GB RAM, and was fitted with an AMD Dual Radeon RX 7600 XT OC Edition 16GB GDDR6 Graphics Card. The display was a Samsung S95CA 77-inch OLED display set to a 144-Hz refresh rate.

For our main analysis, we used an EyeLink 1000+ desktop-mounted eye tracker with a sampling rate of 1000 Hz (unless otherwise stated). EyeLink eye trackers are popular in behavioral research, and by default, they are configured to estimate gaze position by computing the difference between the center of the pupil and the corneal reflection generated by an infrared (IR) illuminator (pupil-CR mode). The camera shipped with EyeLink eye trackers has a detachable infrared filter, which helps to remove nuisance reflections produced by other light sources. However, commercial displays like ours have little emissive power in the infrared range. For this reason, we removed the infrared filter to improve artificial pupil detectability (eye tracker signal-to-noise ratio). Some eye trackers have integrated infrared filters that cannot be removed, but our tests suggest that these systems may work with our method. We return to this issue in the Discussion.

While it is possible to draw both an artificial pupil (dark circle) and a corneal reflection (smaller white circle), for simplicity, we disabled pupil-CR mode, and enabled pupil-only tracking (latency estimates were the same across both modes – see Figure S1). Thus, for all reported analyses, gaze data are estimated based on pupil position alone. Pupil-center was estimated using the ‘centroid’ method (rather than the ‘ellipse-fitting’ method). The sample filter, which can add an additional delay of one to two samples, was switched off.

For our setup, we placed a small mirror on a tripod, near where the observer’s head would typically rest, and positioned the eye tracker to view the display through the mirror (see Fig. 2A). This is not a necessary step, however, as one could just point the eye tracker directly at the display to do the measurements. The reason we use the mirror is to demonstrate that the experimenter does not necessarily need to move/rotate the eye tracker to apply this method (a constraint for eye trackers mounted to the chinrest or desk/table). The Supplementary Materials contain a video of our setup in action (Supplementary Video 1).

Custom software was written in MATLAB with Psychtoolbox (Brainard, 1997). The script used to run all the tests reported in this paper, along with the data reported herein, is available online: https://github.com/mattanderson94/Measure-Gaze-Contingent-Latency. The ‘host’ computer (that controls the eye tracker) was connected to the ‘display’ computer via a direct peer-to-peer Ethernet connection, and the ‘display’ computer was connected to the monitor using an HDMI 2.1b cable.

**Measuring system latency.** To apply our method, the experimenter must first position the eye tracker to clearly view two appropriately scaled pupils (i.e., laterally displaced black circles) rendered on the computer display (see Fig. 2A). While the eye tracker is recording the position of both pupils, one pupil – we will call this the first pupil – moves up and down in a pre-programmed, deterministic trajectory. We achieved good tracking stability with a pupil 0.8 degrees in diameter, that moved 3 degrees per second, with an amplitude of 1 degree (see Fig. 4A for example eye-trace). The second pupil’s horizontal position is fixed, but the vertical position is directly assigned based on the measured position of the first pupil. Some eye trackers have sampling rates that exceed the refresh rates of commercial computer monitors. This results in multiple measurements of the same on-screen gaze position. For example, in this setup, our 144-Hz monitor is sampled at 1000 Hz by the eye tracker, resulting in approximately seven samples per on-screen stimulus. For simplicity, we draw the second pupil based on the *first* gaze position sample available after the Psychtoolbox *Screen(‘Flip’,…)* command that instructs the graphics card to refresh the display with a new frame. While the second pupil is being continuously updated based on the measured position of the first pupil, both pupils are recorded by the eye tracker, and the data are saved for offline analysis. In the tests reported here, the recording lasted 1 min.

We quantify latency in two separate analyses: first, for each sample, we find the time-lag at which the first pupil has a matching vertical position in the second pupil. Second, we correlate the left and right eye traces for a range of time-lags to locate the maximum of the cross-correlation function. The first analysis is useful for estimating the variance of individual updates, and the second analysis gives a single latency estimate that is more robust to lower eye tracker sampling rates.

## Results

**Fig. 3:**
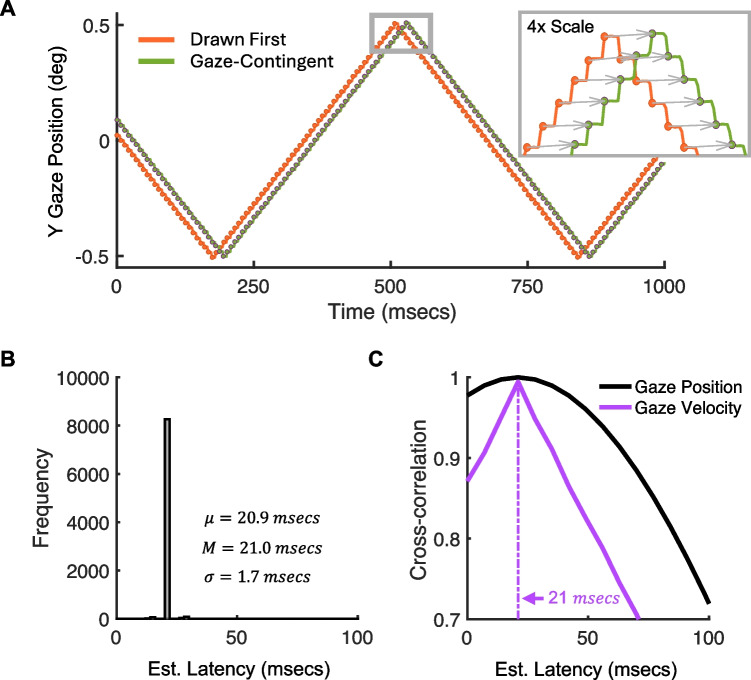
Latency measurements using the proposed method. **A** Vertical position plotted over time for both pupils. The circular markers indicate the first sample made during a ‘flat period’, where multiple measurements are made of the same on-screen pupil position. These are the samples used for our analyses. **B** Histogram of estimated latencies. $M$ gives the median, $\mu$ gives the mean and $\sigma$ gives the standard deviation. **C** Cross-correlation between left and right pupils. Position and velocity are analyzed separately, and they both peak at 21 ms, highlighted by the *vertical dashed line*

Figure 3 presents example results obtained with our method. The vertical position of the left and right pupils, plotted over time, produces a triangular waveform where the second gaze-contingent pupil is temporally delayed relative to the first pupil (Fig. 3A). The lines show every gaze sample, and the circular markers highlight the first sample after each stimulus was updated on the display. The inset shows a zoomed-in view of a small subset of gaze samples. Observe that, because the eye tracker sampling rate exceeds the refresh rate of the monitor, many samples of the same pupil position are made, and this produces a characteristic staircase pattern. The arrows in the inset indicate pairs of samples measured at the same gaze position. The length of these arrows illustrates the time delay between updates. Note that these matches are only approximate, since there is measurement noise in the eye tracker and imperfect vertical alignment of the two pupils on the camera sensor. To apply our analyses, we need not use every gaze sample, since most samples are replicates of the same gaze position. We only take the first sample after each stimulus update. This time reflects how long it took between a change in the first pupil and the corresponding change in the second gaze-contingent pupil. Figure 3B shows the distribution of time delays between pairs of matching left-right gaze positions (arrows in Fig. 3A). The median latency ($M$) is 21.0 ms, and the mean latency ($\mu$) is 20.9 ms which is approximately three frames at 144 Hz. The standard deviation ($\sigma$) is 1.7 ms, indicating low measurement variance. This finding is replicated in our second analysis, where we cross-correlate the left and right pupil signals of eye position over time. Figure 3C plots these data for raw gaze position (black) and gaze velocity (purple). Both variables produce a global maximum at 21 ms.

**Fig. 4:**
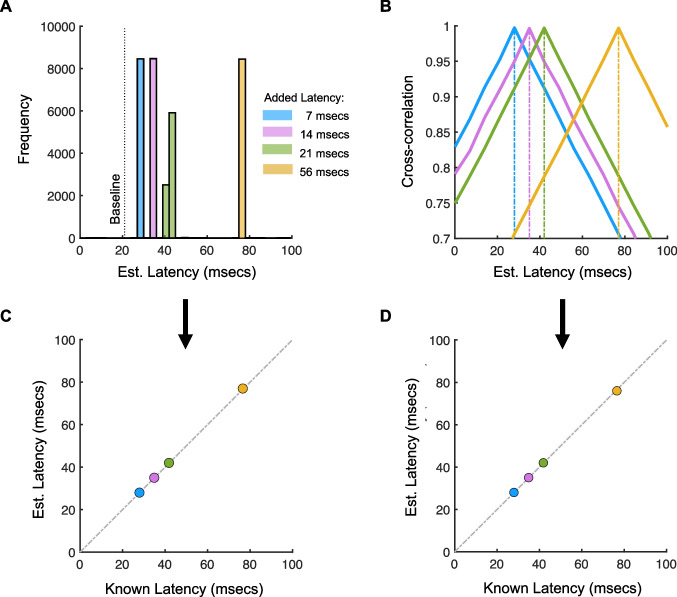
Agreement between estimated and known system latencies. **A** Histogram of measured latencies for various amounts of additional latency that was added programmatically. **B** Cross-correlation between left and right pupil velocities over a range of latencies. Position data are omitted, but produced the same maxima. **C** Median estimated latencies, based on the distributions in **A**, as a function of known system latency. The *dashed diagonal line* indicates perfect measurement accuracy. Standard deviation *error bars* are plotted, but are smaller than the markers. **D** Time delays in B that produced the cross-correlation maxima, plotted as a function of known system latency

The measured latency of ~ 21 ms falls within the range of estimates obtained from existing methods (~ 18–22 ms) that use similar eye trackers and displays with similar refresh rates (Dorr & Bex, 2011; Saunders & Woods, 2014). While this provides an encouraging sanity check, there are still myriad hardware and software differences between setups that limit comparison. Next, we aimed to validate our method by introducing additional rendering delays, in known amounts, and measuring the accuracy and precision with which our method can recover these delays. The logic of these tests is simple: in our experimental program, the location of the second pupil is determined by the latest eye tracker sample available after the display is updated. If, instead of rendering the second gaze-contingent pupil location based on the most recent sample of the first pupil, we use a sample of the first pupil $\Delta t$ ms in the past, we expect to produce a latency (in the second pupil) that matches the baseline latency (~ 21 ms, see above), plus $\Delta t$. The refresh rate of the monitor limits the latencies we can add. In the following tests, we add ~7 ms (one frame), ~14 ms (two frames), ~21 ms (three frames), and ~56 ms (eight frames) of additional latency. Figure 4A, B compares the latency estimates for these different delays. As expected, increasing the programmed latency produces a corresponding increase in the measured latency. More importantly, however, our latency measurements accurately reproduce the expected latencies in the system (which is calculated as the baseline latency, 21 ms, plus the ‘added latency’). Figure 4C plots the median measured latency as a function of known latency, based on the data in Fig. 4A. Standard deviation error bars are shown in this plot, but are so small as to be obscured by the markers, demonstrating the high precision of our measurements. Figure 4D shows the corresponding data for the latencies that produced the maxima in the cross-correlation analysis. Altogether, these results demonstrate that our method is both accurate and precise in picking up real variations in gaze-contingent latencies.

**Fig. 5:**
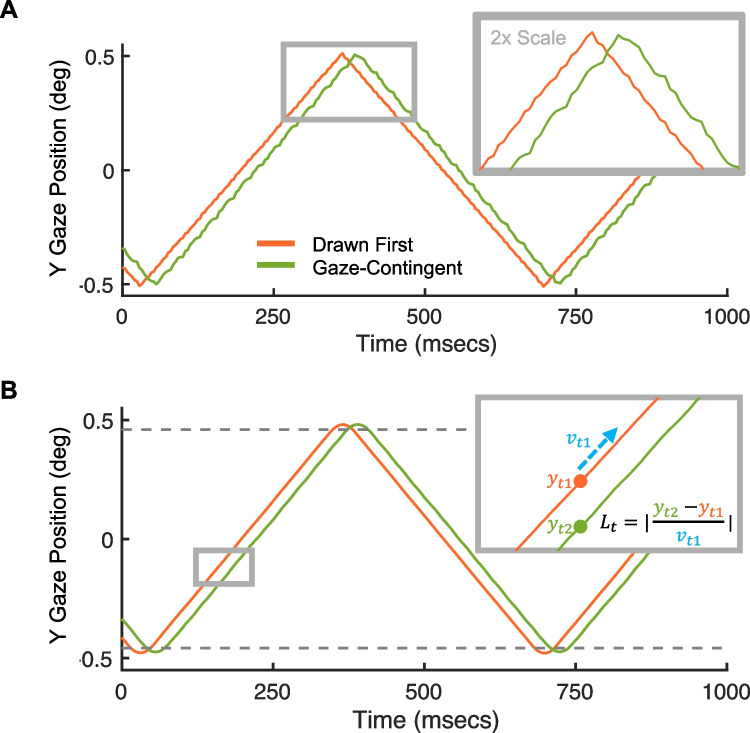
Latency measurements with a lower eye tracker sampling rate (250 Hz). **A** Vertical position plotted over time for both pupils. The inset illustrates the difficulty in isolating individual display updates. **B** The same data in **A**, but smoothed with a mean filter (window size of 40 ms). *Dashed lines* show the 5th and 95th percentiles, which specify the lower and upper bounds for the data included in our analysis. The *inset* shows an illustration of our analysis based on a zoomed-in view of the data. Latency is estimated, for each time-point, based on the *y*-position of the left and right pupils, and the instantaneous velocity of the left pupil

All reported results thus far are based on a 144-Hz monitor refresh rate sampled by the eye tracker at 1000 Hz. In this case, the sampling rate of the eye tracker far exceeds the refresh rate of the monitor, and the discrete time points at which the stimulus was updated can be easily isolated for our analyses. Eye trackers, however, may be operated at (or limited to) sampling rates far below 1000 Hz. Figure 5A shows an example gaze recording for the same pupil stimulus as before, updated at 144 Hz, but sampled at 250 Hz. With this configuration, the eye tracker takes an average of 1.74 measurements per on-screen stimulus. This makes it challenging to estimate latency by locating matching *y*-positions across the left and right pupils, since distinct *y*-positions are harder to segment. This is not an issue for the cross-correlation analysis of estimating latency, but if the experimenter wishes to measure the latency of individual frames, which is useful for quantifying latency *variance*, then we must apply a different analysis.

Assuming that the pupil moves at a constant velocity on the display, we can estimate per-sample latency in a few steps: First, we smooth the raw gaze data using a spatial filter (40-ms mean filter in our case, but other filtering algorithms are common in eye movement research). Second, we compute the estimated latency, $L$, at each sample, $t$, as follows: $L_t=\vert\frac{y_{t2}{-y}_{t1}}{v_{t1}}\vert$ where ${y}_{t1}$ and ${y}_{t2}$ are the vertical positions of the first and second pupil, and *v*_*t*1_ is the vertical instantaneous velocity of the first pupil.

The cartoon inset in Fig. 5B illustrates how this analysis is applied to a single sample. This analysis is only accurate when the velocities of the left and right pupils are approximately equal, which is not true for the brief period when the pupils reverse direction. One simple workaround for this problem is to simply exclude the data around the reversal, which is what we do here. In Fig. 5B, we remove data above and below the 95th and 5th position percentiles, respectively (dashed gray lines). The resulting distribution ($M$= 25.36, $\mu$ = 25.40, $\sigma$=.43) is in general agreement with the results from the cross-correlation analysis (peak at 28 ms). These results demonstrate that, with a small tweak to the applied analysis, our method can be used to measure latency for eye trackers with lower sampling rates. We will consider even lower sampling rates in the Discussion section.

## Discussion

In this paper, we present a convenient, low-cost method of estimating closed-loop, gaze-contingent rendering latencies using a common video-based eye tracker and a commercial display. In tests of our method, we generated high-precision latency measurements that closely aligned with predictions based on the refresh rate of the display.

Obtaining low latency is important in gaze-contingent rendering experiments for several reasons. In research on low vision, for example, gaze-contingent rendering is used to simulate visual field loss in people with normal vision. To study age-related macular degeneration, characterized by central field loss, the experimenter may induce a central scotoma that prevents the observer from processing stimuli around the fovea (Chen et al., 2019; Ganesan et al., 2023; Kwon et al., 2013). Studies that apply this methodology have been used to understand how the brain adapts to visual field loss (Biles et al., 2023; Kwon et al., 2013; Song et al., 2021) and how perceptual learning regimes could be applied to enhance reading speed (Lingnau et al., 2008; Sommerhalder et al., 2004) and visual search (Walsh & Liu, 2014), among other visual functions. In people with real AMD, the scotoma occupies a fixed region of the observer’s visual field. By contrast, an artificial scotoma generated using gaze-contingent rendering will not be perfectly stable, and instability will increase with closed-loop system latency. Motion of the artificial scotoma will result in brief periods in which ostensibly blind regions of the retina are exposed to the visual stimulus. Therefore, the fidelity with which these experiments can simulate visual field loss depends on the latency of gaze-contingent rendering.

No gaze-contingent rendering program can achieve zero latency, so it is natural to ask: how much is too much? One way to answer this question is to measure human detectability of gaze-contingent image manipulations – typically, resolution degradation – for a range of added latencies. Research in this area suggests that latencies greater than ~60 ms produce subjectively noticeable artifacts, although the exact value varies with the spatial scale of the image manipulation (Albert et al., 2017; Loschky & Wolverton, 2007). However, subjective awareness can often diverge from objective measures of functional performance. Returning to our example of artificial scotomas, if the eye moves rapidly from one position to another, and the stimulus is slow to update to the new gaze position, the observer will have a brief glimpse of the stimulus using the region of the visual field that was previously blocked. Humans can encode the identity of masked visual stimuli such as words (Dehaene et al., 2001), objects (Potter et al., 2014), and entire scenes (Anderson et al., 2022), with less than 30 ms of image exposure – far below the ~ 60 ms threshold above. So experimenters should consider whether the rendering latency may offer incomplete control over stimulus placement, and even lead to gaze strategies that exploit slow stimulus updates.

One potential solution to the latency problem is to predict future gaze positions in real time. During fixation, the eye is relatively stable, and rendering latency is less critical for stimulus control, but saccades are high-velocity eye movements that pose a greater problem. Although saccadic suppression limits processing of fine visual details during the saccade itself, when the eye lands at a new location, visual discrimination can be briefly enhanced in a number of ways (Chen & Hafed, 2013; Knöll et al., 2011; Park & Schütz, 2021), which may increase the saliency of latency artifacts. Various researchers have attempted to solve this problem by leveraging gaze velocity statistics to predict saccade landing positions during or before a saccade is executed. Applied methods include predicting landing positions from the first few velocity samples in a saccade (Arabadzhiyska et al., 2017; Han et al., 2013), and using more complex models like recurrent neural networks to predict saccade dynamics from raw gaze position data (Morales et al., 2021). Although these methods are a useful step towards achieving shorter latencies, prediction errors remain an ongoing problem. For instance, Morales et al. (2021) produced an average error rate of approximately 20% the saccade amplitude (the landing position of a 10-degree saccade will be misestimated by about 2 degrees), and other models report larger errors (Arabadzhiyska et al., 2017). This is an unacceptable error size for many applications, and as a result, many researchers continue to update the stimuli based on raw gaze position measurements.

The discussion thus far has highlighted the importance of minimizing latency in gaze-contingent rendering experiments. To optimize our experiments, however, we must first have a way of precisely quantifying latency. Our method is just one of several published approaches, and as such, we present our method not as a replacement for these methods, but as an additional tool. Our method produces similar mean and variance estimates to other studies (Dorr & Bex, 2011; Saunders & Woods, 2014), but precise quantitative comparisons are difficult due to hardware and software differences. Consequently, future research in this area would benefit from a rigorous comparison of these different methods, ideally in a system with a clear ground truth. By extracting multiple latency estimates on the same experimental setup, and quantifying the agreement across methods, researchers can gain a more detailed picture of the costs and benefits of these different approaches, and make an informed judgment about which method to apply. For example, in an experiment where millisecond-level accuracy and precision are needed, a high-speed camera or photodiode may be required, but if the experimenter is less concerned about high precision, a ballpark figure acquired from simply adding up expected delays from the independent components in the system (e.g., eye tracker gaze estimation, frame rendering speeds) may suffice.

In studies that examine the perceptual artifacts created by gaze-contingent latencies, researchers have mainly focused on the impact of mean latency (Albert et al., 2017; Loschky & Wolverton, 2007). Less is known about the effect of latency *variance*. This is a relevant variable in adaptive synchronization display systems (e.g., NVIDIA G-Sync and AMD FreeSync), which continuously monitor the speed of GPU graphics rendering, and adjust the display refresh rate to maximize rendering ‘smoothness’. If an experimental program places a fluctuating load on the GPU by continuously changing image detail/complexity, and this produces a variable refresh rate, then the gaze-contingent rendering latency will also vary over time. The impact of latency variance on perceptual experience is poorly understood, and our method, or similar methods that measure frame-by-frame latency, can be applied to address this question.

Many video-based eye trackers rely on IR emitters and IR imaging to track features of the eye. This can be a problem for our method if the display image does not have sufficient contrast in IR. For our main analysis, we were able to remove the camera IR filters and record the visible light from the display. However, this may not be possible for all systems. We hypothesized that it might be possible to leave the IR filters intact if the display parameters were optimized to maximize IR contrast. Indeed, by showing the on-screen pupils at maximum contrast and increasing display brightness/contrast via the display manufacturer’s software, we were able to reliably track the simulated pupils with the EyeLink 1000+ without removing the IR filters. On OLED displays like the one we used, it also helps to render the white background around the pupils on a small area of the display (due to ‘auto brightness limiting’), and leave the rest of the screen black. All tests were completed in a dark room to minimize screen reflections and ambient light (which also serves to maximize contrast). We also repeated this test using a second eye tracker: a low-cost custom-built eye tracker (Two Blackfly S USB3 04S2M Flir cameras each paired with a 50-mm focal length lens and an IR long-pass filter and an 850-nm IR illuminator, at a cost of ~ $1000 excluding the cost of the computer) running on open-source software OpenIRIS (Sadeghi et al., 2024). This system was also able to track the simulated pupils without any modification to the cameras (see Supplementary Video 2). While we have demonstrated that our method works on commercial eye trackers and inexpensive custom-built eye trackers, it relies on tricking a simple pupil detection algorithm, which can be fooled by rendering false pupils on a 2D screen. Some eye trackers that perform more sophisticated image processing, such as facial recognition and head pose estimation, may be harder to trick. Also, head-mounted eye trackers are not tested here, and may require a complicated system of mirrors (and lenses) to work. We also assume the eye tracker has precise synchronization of the left and right eye recordings. Thus, there are clear limits to the applicability of our method.

Next, we consider how well this method might generalize to eye trackers with even lower sampling rates. For most eye trackers, higher sampling rates are associated with higher shutter speeds, which reduce the time an image is exposed on the camera sensor. As such, lower sampling rates can result in reduced image noise, which may in turn improve the precision of pupil-based gaze estimation, and the precision of our method. However, if the display refresh rate is significantly higher than the eye tracker sampling rate, the eye tracker’s sample may represent an average over multiple refreshes, increasing uncertainty about the estimated gaze position. Additionally, lower sampling rates place a lower bound on the minimum possible system latency. The degree to which this matters depends on the temporal synchrony between the display refresh and the eye tracker sample. If both are well synchronized and the signal delays are small, the eye tracker sampling rate may only need to match the display refresh rate. However, systems such as these are highly sensitive to missing or delayed samples, which are less of a problem for higher sampling rates. Moreover, higher sampling rates have the advantage of allowing the researcher to choose which sample to use, within a given refresh interval (e.g., to apply ‘just-in-time’ sampling). As such, the benefit of low/high eye tracker sampling rates depends on the camera, display, and how the rendering pipeline is programmed.

## Conclusion

Gaze-contingent rendering is a popular technique for investigating how visual processing changes across the visual field. Successful use of this technique, with minimal visual artifacts and experimental confounds, relies on achieving low latencies between gaze measurements and stimulus updates. Consequently, methods of quantifying latency are an important step towards producing high-quality experiments. We describe and test a new method of quantifying latency that does not require additional hardware beyond that which is required to run an eye tracking experiment. We validate our method and demonstrate that it achieves high measurement accuracy and precision, while acknowledging that it may not work for all eye tracking setups. We hope that this method will be a practical tool for quickly and conveniently quantifying the latency of display systems used in gaze-contingent rendering experiments.

## Supplementary Information

Below is the link to the electronic supplementary material.

Supplementary file1 (DOCX 246 KB)

Supplementary file2 (MP4 11.6 MB)

Supplementary file3 (MP4 13.9 MB)

## Data Availability

The datasets generated and analyzed in the current study are available in the GITHUB repository: https://github.com/mattanderson94/Measure-Gaze-Contingent-Latency

## Code availability

All MATLAB scripts used to run our experiments, analyze the resulting data, and produce the figures presented in this paper are available in the GITHUB repository: https://github.com/mattanderson94/Measure-Gaze-Contingent-Latency
