# Supplementary material for: A method for measuring closed-loop latency in gaze-contingent rendering without extra equipment
Source: Behav Res Methods. 2025 Dec 3;58(1):16. doi: 10.3758/s13428-025-02864-3 (PMC12675766; doi:10.3758/s13428-025-02864-3)
Supplement: Supplementary file 1 — Supplementary file1 (DOCX 246 KB) [file 13428_2025_2864_MOESM1_ESM.docx]

Supplementary Materials: Measuring closed-loop latency for gaze-contingent rendering

Figure S1. Latency measurements using the Eyelink ‘pupil-CR’ mode. (A) Vertical position plotted over time for both pupils. The markers indicate the first sample made during a ‘flat period’, where multiple measurements are made of the same on-screen stimulus. These are the samples used for our analyses. (B) Histogram comparing estimated latencies across the Pupil-CR and pupil only modes. *M* indicates the media, $\mu$ the mean and $\sigma$ the standard deviation. (C) Cross-correlation between left and right pupils. Both gaze-tracking methods produced a global maximum at 21 msecs, highlighted by the arrow and vertical dashed line. These data are from a 144 Hz refresh rate sampled by the eye-tracker at 1000 Hz.
